# Hyperthermia Combined with Anti-CTLA-4 Antibody Induces Tumor Microenvironment Remodeling Involving CD4^+^ T Cells in Local and Distant Antitumor Effects in a Murine Triple-Negative Breast Cancer

**DOI:** 10.3390/cancers18081295

**Published:** 2026-04-20

**Authors:** Ayaka Okuuchi, Yoriko Ibuki, Shohei Katsuki, Kazumasa Minami, Shotaro Tatekawa, Keisuke Tamari, Wataru Takenaka, Masahiko Koizumi, Kazuhiko Ogawa, Yutaka Takahashi

**Affiliations:** 1 Department of Medical Physics and Engineering, Graduate School of Medicine, The University of Osaka, Osaka 565-0871, Japan; a_okuuchi@sahs.med.osaka-u.ac.jp (A.O.); yoriko.ibu@sahs.med.osaka-u.ac.jp (Y.I.); katsuki.shouhei.r4y@osaka-u.ac.jp (S.K.); minami.kazumasa.sahs.med@osaka-u.ac.jp (K.M.); takenaka.wataru@sahs.med.osaka-u.ac.jp (W.T.); koizumi.masahiko.sahs.med@osaka-u.ac.jp (M.K.); 2 Department of Radiation Oncology, Graduate School of Medicine, The University of Osaka, Osaka 565-0871, Japan; s_tatekawa.med@osaka-u.ac.jp (S.T.); tamari.keisuke.med@osaka-u.ac.jp (K.T.); ogawa.kazuhiko.med@osaka-u.ac.jp (K.O.); 3 Department of Radiology, Nozaki Tokushukai Hospital, Osaka 574-0074, Japan

**Keywords:** hyperthermia, immune checkpoint inhibitor, tumor microenvironment, abscopal effect, triple-negative breast cancer

## Abstract

**Background/Objectives**: Triple-negative breast cancer (TNBC) is the most aggressive subtype of breast cancer. Our previous study demonstrated that combination therapy with local hyperthermia (HT) and anti-CTLA-4 antibody (C4), an immune checkpoint inhibitor, induced regression of both local and distant tumors. However, tumor microenvironment (TME) changes in local and distant tumors following local HT and C4 remain unclear. Here, we aimed to evaluate TME changes in local and distant tumors induced by local HT + C4 therapy. **Methods**: Murine TNBC cells were inoculated in both legs of male BALB/cAJcl mice, and only one leg was treated with HT (42.5 °C for 20 min). C4 was administered intraperitoneally every 3 days for a total of 3 doses. For CD4^+^ T cell depletion experiments, anti-CD4 antibody (αCD4) was administered intraperitoneally every 3 days for a total of 12 doses. Tumor-infiltrating immune cells in locally heated tumors and unheated distant tumors were analyzed 9 days after the initial treatment. Furthermore, tumor growth in heated tumors and unheated distant tumors under αCD4 administration was evaluated. **Results**: HT + C4 therapy increased the proportion of helper T cells and elevated the ratio of cytotoxic T cells plus helper T cells to myeloid-derived suppressor cells in both heated tumors and unheated distant tumors. The HT + C4 + αCD4 group exhibited significantly larger tumor growth, compared with the HT + C4 group in both heated (*p* < 0.01) and unheated distant tumors (*p* < 0.01). **Conclusions**: These results suggest that combination therapy of HT and C4 favorably modulates the TME. CD4^+^ T cell infiltration may contribute to both local and distant antitumor effects.

## Simple Summary

Triple-negative breast cancer is the most aggressive subtype of breast cancer. We previously demonstrated that a combination of local hyperthermia (HT) and anti-CTLA-4 antibody enhanced the antitumor response in both local and distant tumors. However, it remained unclear how this combination therapy alters the tumor microenvironment (TME). In this study, we examined TME changes in both local and distant tumors in a triple-negative breast cancer mouse model. We found that the combination therapy increased the proportion of helper T cells and decreased immune suppressive myeloid-derived suppressor cells, both in heated and unheated distant tumors. Depletion of CD4^+^ T cells attenuated the antitumor effect of HT + C4 therapy in both heated tumors and unheated distant tumors. These findings suggested that HT + C4 induced TME remodeling involving CD4^+^ T cell infiltration in both local and distant antitumor effects.

## 1. Introduction

Breast cancer is the most frequently diagnosed cancer among women and ranks as the second leading cause of cancer death worldwide [1,2]. Breast cancer has multiple subtypes with different molecular characteristics, among which triple-negative breast cancer (TNBC) accounts for approximately 15% of breast cancer [3,4]. Because TNBC lacks expression of estrogen receptor (ER), progesterone receptor (PR) and human epidermal growth factor receptor 2 (HER2), targeted therapeutic options are limited, resulting in clinical failure in over half of TNBC patients [5,6].

Immune checkpoint inhibitor (ICI), which inhibits immune checkpoints such as programmed cell death protein-1 (PD-1), programmed cell death ligand-1 (PD-L1) and cytotoxic T-lymphocyte-associated antigen-4 (CTLA-4), is an effective treatment for various cancers [7,8,9]. Some reports indicate that CTLA-4 expression is higher in TNBC compared to other breast cancer subtypes [2,10], but monotherapy with ICI often fails to improve outcomes in breast cancer [11,12]. This limited efficacy is partly attributed to the tumor microenvironment (TME) in TNBC, where the abundance and activation/exhaustion states of tumor-infiltrating lymphocytes (TILs) significantly influence treatment efficacy [13,14].

In the immunity cycle, T cells play an important role in antitumor immunity [15,16]. Cytotoxic T lymphocytes (CTL) are activated by recognition of tumor antigens presented by antigen-presenting cells (APC), such as dendritic cells (DC), thereby contributing to the elimination of cancer cells. CTL activation requires a co-stimulatory signal between CD80/86 on APCs and CD28 on CTL, in addition to antigen presentation. However, CTLA-4 inhibits the co-stimulatory signal by binding CD80/86 with higher affinity than CD28, leading to the suppression of CTL activation. Anti-CTLA-4 antibody (C4), an ICI, blocks CD80/86-CTLA-4 binding, thereby enabling CTL to attack tumors [17,18]. Furthermore, CTLA-4 is also expressed on regulatory T cells (Treg), and C4 attenuates Treg-mediated immune suppression [19]. Helper T cells not only enhance CTL activity but also exert cytotoxic effects, highlighting their important roles in antitumor immunity [20,21]. In contrast, myeloid-derived suppressor cells (MDSCs) are known to proliferate within the TME and suppress the function of CTL and helper T cells. Therefore, CTL activation may be partially constrained by the influence of immunosuppressive cells such as MDSCs [22].

Hyperthermia (HT) has long been used in cancer treatment, because of its cell-killing effect obtained by heating tumors to over 42.5 °C [23]. In addition to the direct cell-killing effects, HT induces immunogenic cell death through the release of heat shock proteins [24]. Xu et al. reported that HT reduces TNBC cell survival and promotes TME remodeling [25].

Our previous study demonstrated that combination therapy with local HT and C4 induces regression of both local and distant tumors, known as the abscopal effect, in a TNBC mouse model [26]. However, TME changes in local and distant tumors following local HT and C4 remain unclear. Here, we aimed to evaluate TME changes in local and distant tumors induced by local HT + C4 therapy.

## 2. Materials and Methods

### 2.1. Cell Lines

Murine TNBC cell line (4T1) (RRID: CVCL_0125) was purchased from American Type Culture Collection (Manassas, VA, USA). Briefly, 4T1 cells were cultured in Roswell Park Memorial Institute (RPMI) 1640 (#R0883-500ML, Sigma Aldrich, Tokyo, Japan) with 10% fetal bovine serum (FBS) (#A5256701, Thermo Fisher Scientific, Waltham, MA, USA) and 1% penicillin/L-glutamine (#06168-34, Nacalai Tesque, Kyoto, Japan) at 37 °C and 5% CO_2_.

### 2.2. Hyperthermia

**Figure 1:**
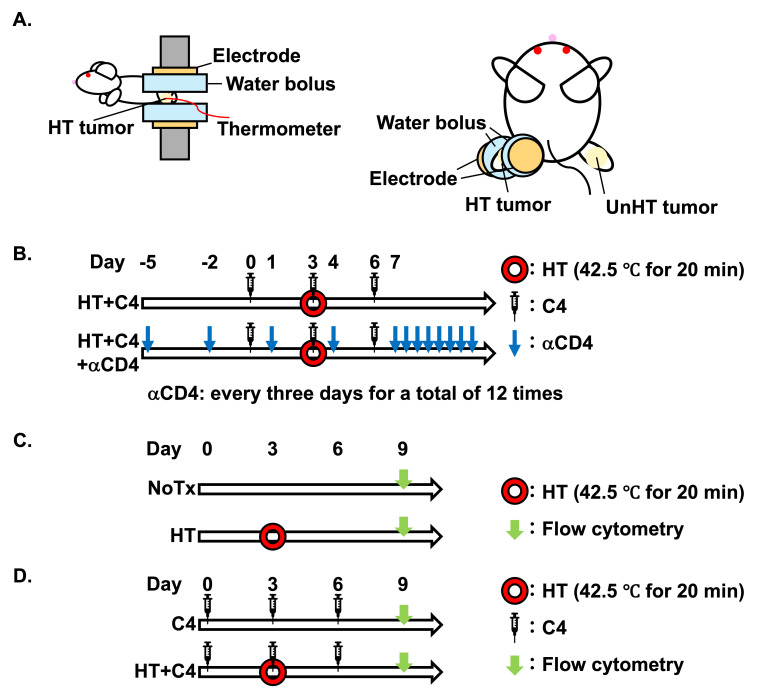
Experimental setup and design. (**A**) Lateral and top views of the in vivo experimental setup. A flexible thermocouple sensor supplied with an HT device was placed on the tumor surface. (**B**) Experimental design for tumor volume changes in the HT + C4 and HT + C4 + αCD4 groups. (**C**) Experimental design for analysis of TME by flow cytometry in the NoTx and HT groups, and (**D**) in C4 and HT + C4 groups. Abbreviations: HT: hyperthermia, C4: anti-CTLA-4 antibody, αCD4: anti-CD4 antibody, NoTx: no treatment, TME: tumor microenvironment.

HT was conducted by an HT device using a radiofrequency (RF) pulse of 8 MHz (YAMAMOTO VINITA, Osaka, Japan). To treat the mice with HT, only one side of the legs was placed between two electrodes through water boluses, while the temperature was monitored using a flexible thermocouple sensor supplied with the HT device (Figure 1A). Water boluses (#JP-65, diameter: 16 mm, thickness: 16 mm) were filled with 3% saline, which is electronically conductive and enables efficient heating while preventing heat generation within the bolus itself. The conductivity of the solution was approximately 45–55 mS/cm. A flexible thermocouple sensor supplied with the HT device was placed beneath the water bolus, corresponding to the tumor surface. Because the water bolus itself does not generate heat, the measured temperature reflects the tumor temperature. All treatments were conducted at 42.5 °C for 20 min. Mice in the NoTx and C4 groups were not immobilized by the in-house jig because the jig itself was not sufficiently restrictive to impair blood flow. Sham-heating was not performed in the NoTx and C4 groups because positioning the tumor-bearing leg between room-temperature water boluses without HT would have exposed the tumor to a cooling condition different from the physiological baseline. The temperature of the body and unheated distant tumors of mice were monitored using a non-contact thermometer (COLEMETER, Tokyo, Japan), and we confirmed that the aiming temperature was achieved only in the heated tumors.

### 2.3. Reagents

C4 (#BE0131, Clone: 9H10, RRID: AB_10950184) purchased from BioXCell (Lebanon, NH, USA) was used. C4 at 150 μg diluted with 100 μL of phosphate-buffered saline (PBS) (#10010023, Thermo Fisher Scientific) was intraperitoneally injected on Days 0, 3, and 6. Anti-CD4 antibody (αCD4) (#BE0003-1, Clone: GK1.5, RRID: AB_1107636) purchased from BioXCell (Lebanon, NH, USA) was used and 150 μg of αCD4 diluted with 100 μL of PBS was intraperitoneally injected every 3 days from Day −5 for a total of 12 doses (Figure 1B). We selected this dosing regimen assuming substantial depletion of CD4^+^ T cells based on a previous study demonstrating that αCD4 administration of 200 µg on Days 5 and 9 effectively induced CD4^+^ T cell depletion [27]. The present study used a greater cumulative dose administration over a long period, compared with the previous study.

### 2.4. Animal Experiments

Six to eight-week-old male BALB/cAJcl mice (RRID: IMSR_JCL:JCL:MIN-0005) were purchased from Nihon-Clea (Tokyo, Japan). Both legs of mice were subcutaneously inoculated with 1 × 10^5^ cells of 4T1 diluted with 60 μL of PBS. Mice were maintained in a pathogen-free animal breeding room with three mice per cage at the University of Osaka, and mice were acclimatized for at least one week.

All protocols for animal experiments were approved by the University of Osaka Institutional Animal Care and Use Committee (Approved#: 30-013-004) following the principles and procedures outlined in the Japanese Act on the Welfare and Management of Animals and Guidelines for the Proper Conduct of Animal Experiments issued by the Scientific Council of Japan. In the survival experiments, mice were monitored at least every other day and sacrificed humanely with CO_2_ over-inhalation when a longer diameter of the tumor reached ≥20 mm, or they met the following criteria: difficulty in breathing, epistaxis, or rotation. Buprenorphine was used when mice experienced unbearable pain.

For local HT, mice were immobilized on an in-house jig under anesthesia. Mice were anesthetized by intraperitoneal injection of Medetomidine hydrochloride (0.75 mg/kg), Midazolam (4 mg/kg), and Butorphanol tartrate (5 mg/kg). Anesthesia was maintained for approximately 30 min. After the experiment, anesthesia was reversed by intraperitoneal injection of Atipamezole (0.75 mg/kg). Mice in the NoTx and C4 groups were also anesthetized for approximately 30 min and then recovered in the same manner as the heated mice. The mice were randomly allocated to the different treatment groups. Potential confounders such as the order of treatments and measurements, or animal/cage location, were not controlled. However, tumor volume measurements were conducted in a blinded manner, whereas other data analysis was not blinded.

### 2.5. Flow Cytometry

The proportions of tumor-infiltrating immune cells in locally heated tumors and unheated distant tumors in the no treatment (NoTx), HT, C4, and HT + C4 groups were evaluated on Day 9 after the initial treatment, corresponding to Day 6 after HT (Figure 1C,D). This time point was selected based on a previous report, demonstrating that CD8^+^ T cell-dependent antitumor effects were observed 7 days after HT [28]. In addition, the sample size was determined based on previous studies [29]. Variation in sample size among experiments was due to technical and practical limitations (e.g., insufficient yield of single-cell suspensions from harvested tumors). We confirmed that there were no differences in the volume of heated tumors or unheated distant tumors on Day 0 between the NoTx and HT groups or between the HT and HT + C4 groups. Minced tumors were incubated in FACS buffer (2% FBS and 0.5 mM EDTA) supplemented with 1 mg/mL collagenase IV (#C4-28, Sigma Aldrich) and 0.2 mg/mL DNase (#10104159001, Sigma Aldrich) at 37 °C on a shaker for 20 min. After Fc block using CD16/32 antibody (#101320, BioLegend, San Diego, CA, USA, RRID: AB_1574975) for 10 min at room temperature, isotype control-FITC (#11-4321-41, Clone: eBR2a, eBioscience, San Diego, CA, USA, RRID: AB_10669560), isotype control-APC (#17-4321-81, Clone: eBR2a, eBioscience, RRID: AB_470181), and isotype control-PE (#12-4321-42, Clone: eBR2a, eBioscience, RRID: AB_1518773), CD45-FITC (#11-0451-82, Clone: 30-F11, eBioscience, San Diego, CA, USA, RRID: AB_465050), CD8a-APC (#17-0081-82, Clone: 53-6.7, eBioscience, RRID: AB_469335), CD4-APC (#17-0042-82, Clone: RM4-5, eBioscience, RRID: AB_469323), CD11b-APC (#17-0112-82, Clone: M1/70, eBioscience, RRID: AB_469343) or Ly-6G/Ly-6C-PE (#14-5931-82, Clone: RB6-8C5, eBioscience, RRID: AB_467730) antibodies were added and reacted for 30 min on ice.

For intracellular staining with Granzyme B (GzmB) or FoxP3, the FoxP3 staining kit (#00-5523-00, eBioscience) was used for fixation and permeabilization according to the manufacturer’s instructions. Then cells were reacted with GzmB-PE (#12-8898-82, Clone: NGZB, eBioscience, RRID: AB_10870787) or FoxP3-PE (#14-5773-82, Clone: FJK-16s, eBioscience, RRID: AB_465936) antibodies for 30 min on ice to evaluate the proportion of CTL or Tregs, respectively. The CD45-FITC antibody was diluted in a ratio of 1:100, and the other antibodies were diluted 1:80. The stained cells were analyzed using the FACS Verse (BD, Franklin Lakes, NJ, USA) and Flow Jo ver. 10 software (Tommy Digital Biology, Tokyo, Japan, RRID: SCR_008520).

### 2.6. Tumor Volume Study

Tumor volume was measured at least every 3 days and calculated by the following formula: V = L × S^2^ × 0.52, where V, L, and S represent tumor volume, the longest length, and the width perpendicular to L, respectively. Relative tumor volume was calculated by normalizing the tumor volume to the volume on Day 0, when C4 treatment was initiated.

### 2.7. Statistics

The statistical significance between the two groups of flow cytometry experiments and tumor volume was tested by the Wilcoxon rank-sum test due to the small number of samples. Because the flow cytometric analysis was exploratory, *p*-values were not adjusted for multiple comparisons among the different immune cell populations. All statistical analyses were conducted using JMP^®^ 18.2.1 (JMP Statistical Discovery LLC., Cary, NC, USA, RRID: SCR_022199).

## 3. Results

### 3.1. Immune Microenvironment Alterations in Heated Tumors

**Figure 2:**
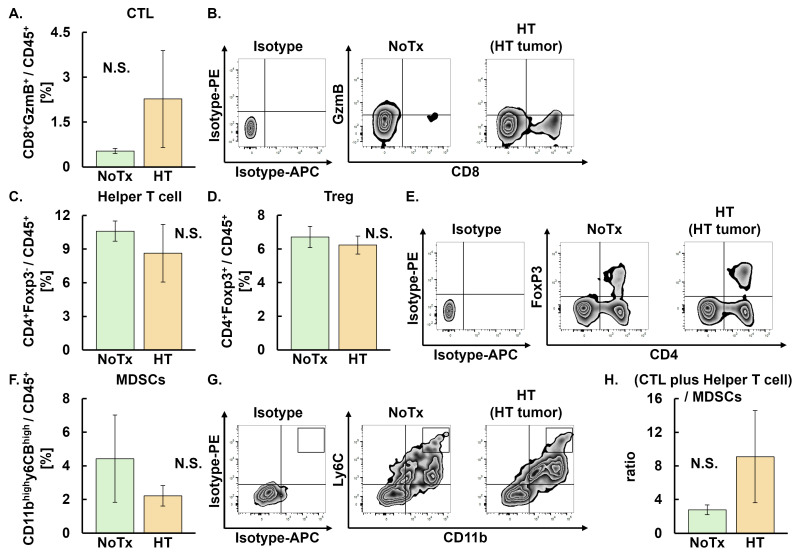
Evaluation of tumor-infiltrating immune cells following HT in heated tumors on Day 9 in the NoTx and HT groups. Error bars represent SEM (*n* = 10 in the NoTx, and *n* = 8 in the HT groups). A total of 18 mice were used. (**A**) Population of CTL: NoTx, 0.53 ± 0.09 [−1.64, 2.71]; HT, 2.27 ± 1.73 [−0.16, 4.71]. (**B**) Representative zebra plots of CTL. (**C**) Population of helper T cells: NoTx, 10.60 ± 2.74 [5.23, 15.97]; HT, 8.63 ± 2.58 [2.21, 15.05]. (**D**) Populations of Treg: NoTx, 6.71 ± 0.66 [5.44, 7.98]; HT, 6.23 ± 0.58 [4.71, 7.75]. (**E**) Representative zebra plots of helper T cells and Treg. (**F**) Populations of MDSCs: NoTx, 4.43 ± 2.21 [2.59, 6.26]; HT, 2.21 ± 0.70 [−0.04, 4.46]. (**G**) Representative zebra plots of MDSCs. (**H**) Ratios of CTL plus helper T cells to MDSCs: NoTx, 2.78 ± 0.64 [1.13, 4.42]; HT, 9.10 ± 6.33 [−11.06, 29.25]. Data are presented as mean ± SEM [95% CI]. Abbreviations: HT: hyperthermia, NoTx: no treatment, CTL: cytotoxic T lymphocyte, Treg: regulatory T cell, MDSCs: myeloid-derived suppressor cells, N.S.: not significant, SEM: standard error of mean, CI: confidence interval.

Our group previously demonstrated that local HT alone did not induce the abscopal effect, whereas the combination of HT and C4 induced the abscopal effect using a bilateral TNBC subcutaneous mouse model [26]. Based on these findings, we first evaluated whether monotherapy with local HT altered the TME. No differences were observed between the NoTx and HT groups in the proportion of CTL (CD45^+^CD8^+^GzmB^+^), helper T cells (CD45^+^CD4^+^FoxP3^−^), Treg (CD45^+^CD4^+^FoxP3^+^), and MDSCs (CD45^+^CD11b^high^Ly6c^high^). Accordingly, the ratio of CTL plus helper T cells to MDSCs did not differ between the two groups in heated tumors (Figure 2A–H).

**Figure 3:**
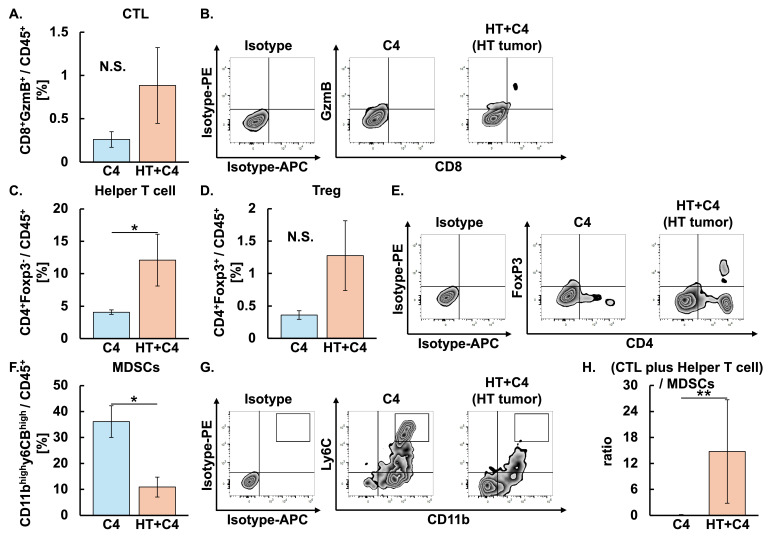
Evaluation of tumor-infiltrating immune cells following HT + C4 therapy in heated tumors on Day 9 in the C4 and HT + C4 groups. Error bars represent SEM (*n* = 6 in the C4, and *n* = 6 in the HT + C4 groups). A total of 12 mice were used. (**A**) Populations of CTL: C4, 0.26 ± 0.10 [−0.51, 1.03]; HT + C4, 0.88 ± 0.48 [0.11, 1.66]. (**B**) Representative zebra plots of CTL. (**C**) Populations of helper T cells: C4, 4.09 ± 0.38 [−2.10, 10.27]; HT + C4, 12.10 ± 4.47 [5.32, 18.87]. (**D**) Populations of Treg: C4, 0.36 ± 0.07 [−0.48, 1.20]; HT + C4, 1.28 ± 0.60 [0.36, 2.20]. (**E**) Representative zebra plots of helper T cells and Treg. (**F**) Populations of MDSCs: C4, 36.12 ± 6.70 [23.64, 48.59]; HT + C4, 10.92 ± 4.21 [−1.55, 23.39]. (**G**) Representative zebra plots of MDSCs. (**H**) Ratios of CTL plus helper T cells to MDSCs: C4, 0.15 ± 0.03 [0.07, 0.23]; HT + C4, 14.77 ± 13.37 [−22.34, 51.88]. * *p* < 0.05, ** *p* < 0.01. Data are presented as mean ± SEM [95% CI]. Abbreviations: HT: hyperthermia, CTL: cytotoxic T lymphocyte, Treg: regulatory T cell, MDSCs: myeloid-derived suppressor cells, N.S.: not significant, SEM: standard error of mean, CI: confidence interval.

We next investigated whether the combination of C4 and local HT altered the TME in heated tumors. Although the proportion of CTL appeared higher in the HT + C4 group than in the C4 group, the difference did not reach statistical significance (Figure 3A,B). In contrast, the proportion of helper T cells in the HT + C4 group was 3-fold higher than that in the C4 group (*p* = 0.023). The proportion of Treg in the HT + C4 group was comparable to that in the C4 group (Figure 3C–E). Notably, MDSCs in the HT + C4 group decreased by 70% compared with the decrease in the C4 group (*p* = 0.013) (Figure 3F,G). Consequently, the ratio of CTL plus helper T cells to MDSCs was increased in the HT + C4 group compared with the C4 group (*p* = 0.0081), suggesting that the combination of HT with C4 shifted the TME toward an antitumor immune state (Figure 3H).

### 3.2. Immune Microenvironment Alterations in Unheated Distant Tumors

**Figure 4:**
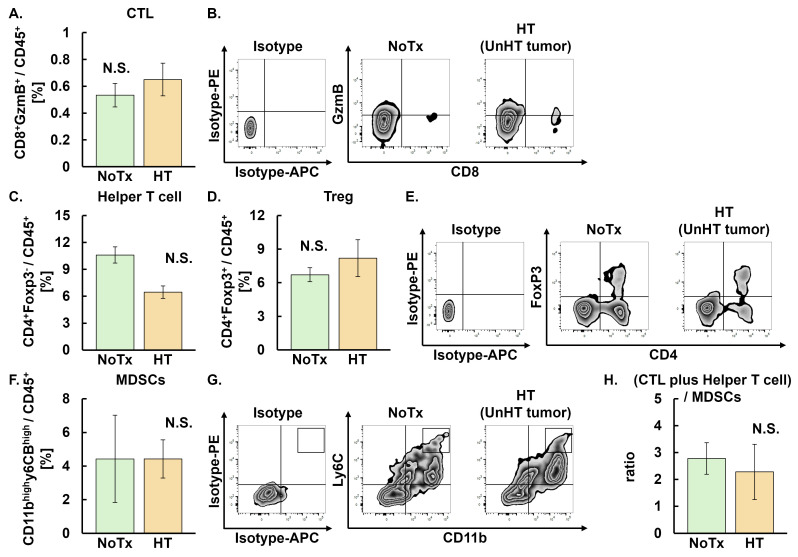
Evaluation of tumor-infiltrating immune cells following HT therapy in unheated distant tumors on Day 9 in the NoTx and HT groups. Error bars represent SEM (*n* = 10 in the NoTx, and *n* = 7 in the HT groups). A total of 18 mice were used. One mouse was excluded from the analysis due to an insufficient yield of single-cell suspensions from the harvested tumor. (**A**) Populations of CTL: NoTx, 0.53 ± 0.09 [0.32, 0.75]; HT, 0.65 ± 0.13 [0.40, 0.90]. (**B**) Representative zebra plots of CTL. (**C**) Populations of helper T cells: NoTx, 10.60 ± 2.74 [5.71, 15.49]; HT, 6.47 ± 1.35 [0.15, 12.78]. (**D**) Populations of Treg: NoTx, 6.71 ± 0.66 [4.58, 8.84]; HT, 8.20 ± 1.81 [5.45, 10.94]. (**E**) Representative zebra plots of helper T cells and Treg. (**F**) Populations of MDSCs: NoTx, 4.43 ± 2.21 [2.53, 6.32]; HT, 4.42 ± 0.80 [2.10, 6.73]. (**G**) Representative zebra plots of MDSCs. (**H**) Ratios of CTL plus helper T cells to MDSCs: NoTx, 2.78 ± 0.64 [1.13, 4.42]; HT, 2.28 ± 1.19 [−1.51, 6.07]. Data are presented as mean ± SEM [95% CI]. Abbreviations: HT: hyperthermia, NoTx: no treatment, CTL: cytotoxic T lymphocyte, Treg: regulatory T cell, MDSCs: myeloid-derived suppressor cells. N.S.: not significant, SEM: standard error of mean, CI: confidence interval.

As an abscopal effect was observed in the HT + C4 group [26], we next investigated TME differences between NoTx and HT groups in the unheated distant tumors. No differences were observed between the NoTx and HT groups in the proportion of CTL, helper T cells, Treg, or MDSCs and the ratio of CTL plus helper T cells (Figure 4A–H).

**Figure 5:**
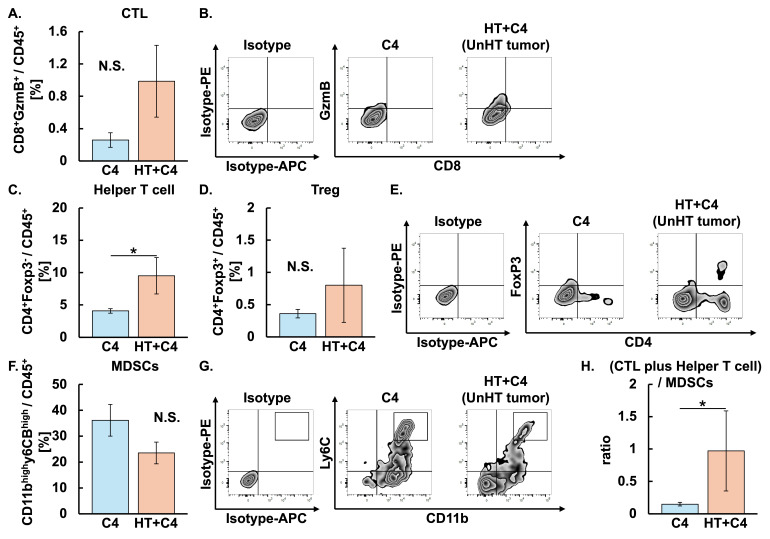
Evaluation of tumor-infiltrating immune cells following HT + C4 therapy in unheated distant tumors on Day 9 in the C4 and HT + C4 groups. Error bars represent SEM (*n* = 6 in the C4, and *n* = 5 in the HT + C4 groups). A total of 12 mice were used. A mouse was excluded from the analysis due to the insufficient yield of single-cell suspensions from the harvested tumor. (**A**) Populations of CTL: C4, 0.26 ± 0.10 [−0.45, 0.96]; HT + C4, 0.99 ± 0.50 [0.21, 1.76]. (**B**) Representative zebra plots of CTL. (**C**) Populations of helper T cells: C4, 4.09 ± 0.38 [0.72, 7.46]; HT + C4, 10.01 ± 2.40 [0.33, 2.29]. (**D**) Populations of Treg: C4, 0.36 ± 0.07 [−0.54, 1.26]; HT + C4, 1.31 ± 0.64 [0.32, 2.29]. (**E**) Representative zebra plots of helper T cells and Treg. (**F**) Populations of MDSCs: C4, 36.12 ± 6.70 [23.12, 49.11]; HT + C4, 23.54 ± 4.67 [9.30, 37.78]. (**G**) Representative zebra plots of MDSCs. (**H**) Ratios of CTL plus helper T cells to MDSCs: C4, 0.15 ± 0.03 [0.07, 0.23]; HT + C4, 0.99 ± 0.69 [−0.92, 2.89]. * *p* < 0.05. Data are presented as mean ± SEM [95% CI]. Abbreviations: HT: hyperthermia, CTL: cytotoxic T lymphocyte, Treg: regulatory T cell, MDSCs: myeloid-derived suppressor cells, N.S.: not significant, SEM: standard error of mean, CI: confidence interval.

We next examined differential TME in the unheated distant tumors between the C4 and HT + C4 groups to determine whether TME alteration contributed to the abscopal effect. Although the proportion of CTL appeared to be higher in the HT + C4 group than in the C4 group, the difference was not significant (Figure 5A,B). The proportion of helper T cells increased 2.3-fold in the HT + C4 group compared with that in the C4 group (*p* = 0.014). The proportions of Treg and MDSCs in the HT + C4 were comparable between the two groups (Figure 5C–G). However, the ratio of CTL plus helper T cells to MDSCs in the HT + C4 group was 6.6-fold higher than that in the C4 group (*p* = 0.0225) (Figure 5H). Specifically, the ratio of CTL plus helper T cells to MDSCs in the C4 and HT + C4 groups was 0.15 ± 0.03 and 0.99 ± 0.69, respectively.

### 3.3. Depletion of CD4^+^ T Cells Diminishes the Efficacy of HT + C4 Therapy

**Figure 6:**
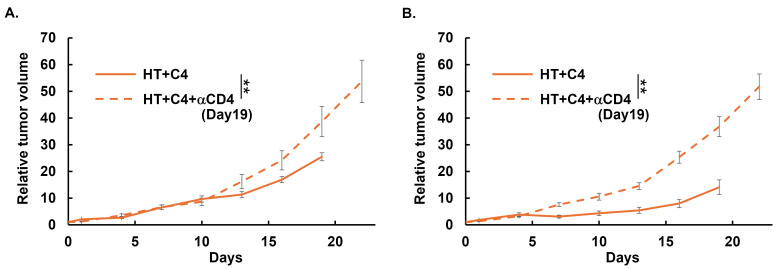
Tumor volume changes in HT + C4 and HT + C4 + αCD4 groups. Relative tumor volume was calculated by normalizing the tumor volume to the tumor volume on Day 0, when C4 treatment was initiated. Error bars represent SEM (*n* = 10 in the HT + C4, and *n* = 10 in the HT + C4 + αCD4 groups). A total of 20 mice were used. The sample size was determined based on a previous study [27]. (**A**) Heated tumor volume changes. On Day 19, when a significant difference was observed, the mean tumor volume was 25.51 ± 1.47 in the HT + C4 group and 38.66 ± 5.67 in the HT + C4 + αCD4 group (mean ± SEM; 95% CI, HT + C4: [16.81, 34.22], HT + C4 + αCD4: [29.96, 47.37]). (**B**) Unheated distant tumor volume changes. On Day 19, when a significant difference was observed, the mean tumor volume was 14.12 ± 2.75 in the HT + C4 group and 36.75 ± 3.75 in the HT + C4 + αCD4 group (mean ± SEM; 95% CI, HT + C4: [7.21, 21.03], HT + C4 + αCD4: [29.85, 43.66]). ** *p* < 0.01. Abbreviations: HT: hyperthermia, C4: anti-CTLA-4 antibody, αCD4: anti-CD4 antibody, SEM: standard error of mean, CI: confidence interval.

We previously reported that HT + C4 therapy enhanced both local control and the abscopal effect, compared with monotherapy with C4 [26]. In the present study, we showed that the proportion of helper T cells (CD45^+^, CD4^+^, FoxP3^−^) was increased in TME in both heated tumors and unheated distant tumors, whereas no significant changes were observed in Treg (CD45^+^, CD4^+^, FoxP3^+^). Therefore, we investigated whether helper T cells contribute to tumor growth by comparing the HT + C4 group and the HT + C4 + αCD4 group. Depletion of CD4^+^ T cells in the HT + C4 + αCD4 group resulted in significantly increased tumor volume in both heated tumors (*p* = 0.0091) and unheated distant tumors (*p* = 0.0010) compared to the HT + C4 group (Figure 6A,B), suggesting that CD4^+^ T cells contribute to the treatment efficacy of HT + C4 treatment in both heated and unheated distant tumors. No side effects were observed in both groups. All mice were euthanized due to the humane endpoint (tumor size exceeding 20 mm in the longest diameter), rather than due to the metastases.

## 4. Discussion

We previously demonstrated that HT monotherapy failed to induce either local control or abscopal effect, whereas the addition of C4 to HT successfully induced both effects. Furthermore, the abscopal effect induced in the HT + C4 group disappeared with the administration of FTY720, a sphingosine-1-phosphate receptor modulator that prevents lymphocyte trafficking from lymphoid organs to peripheral tissues, suggesting that the abscopal effect is attributed to lymphocyte trafficking from lymphoid organs to tumors [26]. However, the specific immune cell population in the TME that mediates the abscopal effect has remained unclear.

We evaluated the tumor-infiltrating immune cells from heated tumors and unheated distant tumors to investigate whether local HT changes TME. A previous report using a 4T1 mouse model demonstrated that the increases in CD4^+^ and CD8^+^ T cell infiltrations into the tumor peaked at 24 h after RF-based local HT at 41 °C for 30 min. These increased levels returned to baseline within 48 h due to the increased proportion of MDSCs, which impaired T cell trafficking and limited antitumor efficacy [30]. Similarly, our data showed no significant alteration in populations of tumor-infiltrating immune cells by local HT monotherapy in either heated tumors or unheated distant tumors 6 days after local HT. In contrast, Hu et al. reported that intracellular aggregation of magnetic particles enhanced HT efficiency and immune activation in 4T1 mice model [31]. Moreover, another report using the 4T1 mice model showed that iron oxide nanoparticle-mediated HT induced TME changes, where infiltration of antigen-presenting cells and expression of heat shock proteins were increased, thereby augmenting antitumor immunity [32]. These findings suggest that the extent of immune activation induced by HT depends on the heating methods and heating temperature, as nanoparticle-mediated HT produces a higher temperature than RF-based HT.

Because the immune microenvironment was not altered by local HT monotherapy in the present study, we next investigated whether the addition of C4 to HT alters the immune microenvironment within the tumor. We found that the proportion of MDSCs decreased, whereas that of helper T cells increased in heated tumors, leading to the increased ratio of CTL plus helper T cells to MDSCs compared with that in C4 monotherapy. The combination of local HT and C4 is considered to promote immune activation through multiple mechanisms. Local HT induces tumor antigen release and upregulation of heat shock proteins and facilitates immune cell infiltration by improving tumor perfusion [33,34,35]. Meanwhile, C4 promotes the activation of cytotoxic immunity [17]. These synergistic effects might contribute to the observed increase in helper T cells and decrease in MDSCs. Indeed, a recent study using the 4T1 mice model demonstrated that combining mild photothermal therapy with anti-PD-L1 antibodies leads to a significant reduction in MDSCs and increased CD8^+^ T cell infiltration, leading to tumor regression of heated tumors and prolonged survival [36]. Although the heating modality and ICI used differed from our study, the findings generally align with our results, suggesting that the combination of HT and ICI contribute to the TME remodeling mediated by immunosuppressive MDSCs.

Importantly, the immune microenvironment was altered not only in heated tumors but also in unheated distant tumors treated with a combination of local HT and C4, in which the proportion of helper T cells increased with a trend toward a higher CTL plus helper T cells to MDSCs ratio than C4 monotherapy. These results align with our previous report demonstrating that HT + C4 treatment induced the abscopal effect [26].

These immune cell analyses were performed in an exploratory manner to broadly characterize distinct aspects of the TME, including CTL, helper T cell, Treg, MDSC, and their ratio. Therefore, we did not apply a formal correction for multiple comparisons because our intent was hypothesis generation rather than confirmatory statistical testing. Accordingly, *p*-values in the flow cytometric analysis should be interpreted cautiously.

Importantly, the increase in CD4^+^ helper T cells identified in this exploratory analysis led us to perform the subsequent CD4^+^ T cell depletion experiment. Our results revealed that in both heated tumors and unheated distant tumors, depletion of CD4^+^ T cells significantly accelerated tumor growth. However, caution is required for the data interpretation. First, we did not include the C4 + αCD4 group. Our previous study showed that C4 monotherapy provided no therapeutic benefit, compared with NoTx in both heated and unheated distant tumors, whereas HT + C4 induced significant antitumor efficacy in both heated and unheated distant tumors [26]. Furthermore, in the present study, we observed an increased proportion of CD4^+^ helper T cells in the HT + C4 group compared with the C4 group. Based on these results, we conducted the CD4^+^ T cell depletion experiment by comparing the HT + C4 and HT + C4 + αCD4 groups. Although the absence of a C4 + αCD4 group prevents us from completely excluding an effect of CD4^+^ T cell depletion on the baseline effect of C4 alone, our previous finding that C4 monotherapy did not provide therapeutic benefit, together with the increased proportion of CD4^+^ T cells observed specifically in the HT + C4 group, suggests that any such contribution is likely minimal. Therefore, the reduced efficacy following CD4^+^ T cell depletion is more likely due to the CD4^+^ T cell compartment of the antitumor response induced by HT + C4. Second, we did not include isotype control antibodies for either C4 or αCD4 treatment. Therefore, we cannot completely exclude the possibility that a part of the observed effects was influenced by non-specific immune responses to the administration of foreign IgG. Although an isotype control for αCD4 treatment was not included, the αCD4 clone GK1.5 has been extensively validated as an in vivo depleting antibody and has been used in previous studies with PBS-treated controls [34]. Therefore, although inclusion of appropriate isotype control antibodies would further strengthen the specificity of our conclusions, the present experimental design still supports the conclusion that the observed differences are primarily attributable to the additional αCD4 treatment rather than to non-specific effects of IgG administration. Third, the αCD4 used in the present study depleted both helper T cells and Treg. Therefore, our data do not definitively distinguish the contribution of these two subsets. Nevertheless, our data suggest that the beneficial effect of the CD4^+^ T cell compartment is required for the antitumor efficacy of HT + C4. However, the specific contribution of individual CD4^+^ T cell subsets (e.g., Th1, Th2, Th17, Tfh, cytotoxic CD4^+^ T cells), as well as other types of immune cells related to innate immunity, such as natural killer cells, macrophages, and DC, or cytokine secretion, remains unclear. Thus, the precise mechanisms by which helper T cells mediate systemic antitumor immunity, including abscopal effects, should be clarified in future studies.

The model used in the present study has several limitations. First, we used male mice. The hormonal and immunological environments of male mice are fundamentally different from those of female mice. However, our primary aim was to evaluate the direct effects of HT with or without C4. Therefore, we used male mice to reduce hormone-related confounding and to understand the clear effects of HT with or without C4, consistent with our previous study [26]. In addition, Albores-Medez et al. reported no significant differences in tumor growth, necrosis, or lung metastasis between male and female mice in the 4T1 model [37]. Nevertheless, the use of female mice to include hormone-related confounding would improve the translational relevance of findings by incorporating hormone-related effects, which should be clarified in future studies. Second, we used a single TNBC mice model. Validation in additional tumor models would strengthen the generalizability of the findings and reduce the possibility that the observed immune responses are model-specific. Third, we used a subcutaneous model rather than an orthotopic model. While a subcutaneous model enables more consistent delivery of hyperthermia to the tumor site, conducting hyperthermia to orthotopically implanted tumors is technically challenging and may introduce variability in temperature control and heating electrode placement. Therefore, the subcutaneous model minimizes such variability, enabling reliable evaluation of the TME changes induced by HT with or without C4. However, an orthotopic model more accurately recapitulates the site-specific TME and may provide findings under more translationally relevant conditions. In addition, the use of a broader range of tumor models, including ICI-responsive models such as E0771, and ICI-resistant models such as AT3 [38] would further enhance the translational relevance of the study. Future studies incorporating hormonal effects on TME in breast cancer models using female mice, orthotopic and multiple tumor models, including both ICI-responsive and ICI-resistant settings, will be important to confirm the robustness and translational relevance of HT with C4.

## 5. Conclusions

These findings suggested that combination therapy with HT and C4 induced TME remodeling involving CD4^+^ T cell infiltration in both local and distant antitumor effects.

## Data Availability

The data presented in this study are available on request from the corresponding author.

## Abbreviations

The following abbreviations are used in this manuscript:

TNBC	Triple-negative breast cancer
ER	Estrogen receptor
PR	Progesterone receptor
HER2	Human epidermal growth factor receptor 2
ICI	Immune checkpoint inhibitor
PD-1	Programmed cell death protein-1
PD-L1	Programmed cell death ligand-1
CTLA-4	Cytotoxic T-lymphocyte-associated antigen-4
TME	Tumor microenvironment
TILs	Tumor-infiltrating lymphocytes
CTL	Cytotoxic T lymphocytes
APC	Antigen-presenting cells
DC	Dendritic cells
C4	Anti-CTLA-4 antibody
Treg	Regulatory T cells
MDSCs	Myeloid-derived suppressor cells
HT	Hyperthermia
RPMI	Roswell Park Memorial Institute
FBS	Fetal bovine serum
RF	Radiofrequency
αCD4	Anti-CD4 antibody
PBS	Phosphate-buffered saline
NoTx	No treatment
GzmB	Granzyme B
